# End-user experiences with two incident and injury reporting systems designed for led outdoor activities - challenges for implementation of future data systems

**DOI:** 10.1186/s40621-019-0214-y

**Published:** 2019-09-09

**Authors:** Caroline F. Finch, Natassia Goode, Louise Shaw, Paul M. Salmon

**Affiliations:** 1 School of Medical and Health Sciences, Edith Cowan University, Joondalup, Perth, Western Australia Australia; 2 Centre for Human Factors and Sociotechnical Systems, University of the Sunshine Coast, Maroochydore, Queensland Australia; 3 College of Science, Health and Engineering, La Trobe University, Melbourne, Victoria Australia

**Keywords:** Injury surveillance, Incident reporting, Data systems, Evaluation, Implementation

## Abstract

**Background:**

Injury and incident (near miss) prevention is heavily dependent upon robust and high-quality data systems. Evaluations of surveillance systems designed to report factors associated with incidents and injuries are essential to understand their value, as well as to improve their performance and efficiency. Despite, this there have been few such evaluations published in the peer-review literature.

**Methods:**

The attitudes and experiences of industry representatives who used one of two variants of an incident and injury surveillance system to collect injury and incident data for the led outdoor activity setting were obtained through an online self-report survey following a 12-month trial. Survey respondents were 18 representatives of 33 organisations who were users of a comprehensive incident reporting and surveillance system – the Understanding and Preventing Led Outdoor Accidents Data System Software Tool (UPLOADS-ST) - and six out of 11 users of a modified system (UPLOADS-Lite). The survey collected information on user experiences in relation to system training, accessibility, ease of use, security, feedback and perceived value to the sector of collating and reporting data across organisations.

**Findings:**

Only four UPLOADS-ST responding users found the system easy to use and just three considered entering incident reports to be easy. However, many considered the training on reporting incidents to be sufficient and that the incident reports contained relevant details. Fewer than half of respondents (seven for UPLOADS-ST, three for UPLOADS-Lite) believed entering data was a good use of staff time and resources. Nonetheless, a majority of respondents (seven for UPLOADS-ST, five for UPLOADS-Lite) found the reporting format easy to read and felt the information provided was useful for their organisation.

**Conclusions:**

Usability barriers to incident reporting were identified, particularly for UPLOADS-ST, including time constraints and user friendliness. The majority of users believed aggregating and reporting incident and injury data across organisations would be of value in making the led outdoor activity sector safer. Improving the utility of the surveillance systems will assist in ensuring their sustainability in the led outdoor activity sector.

## Introduction

Accurate and timely information about injuries and near-miss incidents from data collection and reporting systems is needed to inform the identification and implementation of preventive solutions that can reduce the risk of harm. The perceived relevance and usefulness of such data systems can influence user engagement with them and hence can impact on reporting rates and data quality (Ekegren et al., 2014). The World Health Organization and Centers for Disease Control injury surveillance guidelines (Holder et al., 2001) highlight the quality attributes of good surveillance systems, which include simplicity, flexibility, acceptability, reliability, utility, sustainability and timeliness. The design of any surveillance system and its user interface is critical, as is evaluation of its effectiveness (Mitchell et al., 2009; Bromley et al., 2018). The effectiveness of training and resource materials provided to guide use of the system, and the level of infrastructure and resourcing devoted to data collection and reporting by organisations, are important considerations for data system implementation and sustainability (Kerr et al., 2014; Finch & Staines, 2018). Injury surveillance approaches have long been used in the sports injury settings (Finch, 1997) but have not been widely applied in broader activity settings, especially those not undertaken in traditional sports participation environments.

Led outdoor activities, such as bushwalking, kayaking and abseiling, are defined as facilitated or instructed activities within outdoor education and recreation settings with an associated learning goal. The sector that delivers these activities is both diverse and complex. The number of organisations that deliver such programs is unknown but it is expected to be in Australia’s top 20 sectors of employment (Service Skills Australia, 2015). The organisations themselves range from government bodies to commercial enterprises to not-for-profits (including volunteer organisations). The number of people who participate in such activities under the leadership of a sector employee, either through school programs or outdoor recreation programs, is also unknown but is predicted to be on the increase (Service Skills Australia, 2015). These sector-size limitations also mean that there is currently a paucity of high quality information about the epidemiology and causation of led outdoor activities injuries (Dickson, 2012; Salmon et al., 2010). The Understanding and Preventing Led Outdoor Accidents Data System (UPLOADS) was developed in partnership with key sector representatives to support the collection and reporting of injury and incident data in the led outdoor activity sector in Australia (Salmon et al., 2017; Goode et al., 2018). The system features a reporting form and classification scheme for coding the contributing factors involved in injury, illness, and near miss incidents based around a systems thinking framework (Rasmussen, 1997), so as to provide important information to inform the design and targeting of preventive programs across the Australian led outdoor activity setting (Goode et al., 2018).

Usability of the initial-design of the UPLOADS system was first evaluated after an initial 6-month trial in 2013. In response to some of the trial feedback, which included concerns about system complexity and data entry burden, a modified “Lite” version of the system was developed to reduced data reporting requirements (Goode et al., 2015). Over the following 12-months, led outdoor activities providers and industry representatives were given the option of using either the UPLOADS Software Tool (UPLOADS-ST) or UPLOADS-Lite to record and report on injuries and incidents relating to their organisations. This paper reports the post-implementation evaluation of these users’ experiences in relation to the UPLOADS-ST or UPLOADS-Lite systems. In doing so, it provides useful information to guide the ongoing implementation and refinement of data collection and reporting systems in this setting.

## Methods

The rationale behind, and processes used, to design and define the specific content of the UPLOADS-ST and UPLOADS-Lite systems are published in full elsewhere (Salmon et al., 2017; Goode et al., 2018; Goode et al., 2017; Goode et al., 2016a). UPLOADS-Lite was a modified version of UPLOADS-ST, following feedback received from users of the latter after a 6 month pilot trial (Goode et al., 2015). Table 1 summarises the key design features of the two systems.

**Table 1:** The key design features of the UPLOADS-ST and UPLOADS-Lite injury and incident reporting systems

Features	UPLOADS-ST	Changes made for UPLOADS-Lite and their rationale
IT Platform		
	FileMaker Pro	SurveyMonkey used to collect incident report and Excel spreadsheet to collect participation data. This was in response to an industry need for a very easy, quick, online data-reporting tool that did not require knowledge about a specific program.
Paper-based incident reports		
	Yes	Yes
Collects incident reports		
	Yes	Yes
Collects participation data (for exposure measurement)		
	Yes	Yes – this was through an Excel spreadsheet
Stores incident reports in a format accessible to organization		
	Yes	No. The system was designed as a data repository only for the purposes of collation of information across organisations. It was not designed to provide detailed incident reports for organisations to access, as it was assumed that they already had that data themselves on individual incidents and were not interested in undertaking their own detailed cross-incident analysis.
Stores participation data in a format accessible to organization		
	Yes	Yes
Analyse and classifying contributory factors in incident reports		
	Yes	No. Detailed incident description and analysis, from a safety systems point of view, requires a high level of understanding and expertise. Organisations chose to use UPLOADS-Lite because they were not interested in, or did not have the skills, to do this themselves. These organisations still wanted to provide data about led outdoor activity incidents, without a requirement of also needing to analyse them in detail, which was a key feature of UPLOADS-ST.
Produce aggregate summaries of contributory factors involved in incidents		
	Yes	No. Detailed incident description and analysis, from a safety systems point of view, requires a high level of understanding and expertise. Organisations chose to use UPLOADS-Lite because they were not interested in, or did not have the skills, to do this themselves. These organisations still wanted to still provide data about led outdoor activity incidents, without a requirement of also needing to analyse them in detail, which was a key feature of UPLOADS-ST.
Generate descriptive statistics on the incident data		
	Yes	No. Detailed incident description and analysis, from a safety systems point of view, requires a high level of understanding and expertise. Organisations chose to use UPLOADS-Lite because they were not interested in, or did not have the skills, to do this themselves. These organisations still wanted to still provide data about led outdoor activity incidents, without a requirement of also needing to analyse them in detail, which was a key feature of UPLOADS-ST.
Contribute data to the National Incident Dataset		
	Yes	Yes
Confidentiality of submission of data to the National Incident Dataset		
	Automatically de-identified (names removed) data to send via email to research team. Data was then merged for analysis and organization given a code. Organization was re-identifiable	Submission of de-identified incident reports was completely anonymous. Excel spreadsheets submitted via email. This was to protect the privacy of organisations contributing their incident data to a larger national compilation of incidents.
Training		
	A manual describing the overall approach to incident reporting and analysis, this included the systems thinking approach and how to collect appropriate incident data; A manual describing each component of UPLOADS, including the classification schemes and the Software Tool; A manual describing how to create reports on incident statistics and interpret the Accimap analyses; On-line videos demonstrating how to use the Software Tool (e.g. entering incidents reports, classifying contributory factors and relationships, aggregate analyses)	A manual describing the overall approach to incident reporting and analysis, this included the systems thinking approach and how to collect appropriate incident data and 1 page of instructions for using Survey Monkey and the excel spreadsheet. Because UPLOADS-Lite did not include functionality around detailed interrogation of incident reports and identification of causal factors, no further training was provided.

Both the UPLOADS-ST and UPLOADS-Lite were trialled within organisations identified as being part of the Australian led outdoor activities sector. Organisations were invited to participate via peak body and professional membership association newsletters, and relevant social media pages. Interested organisations were asked to invite a senior staff member in a safety-related role to be the primary contact. This person was responsible for undertaking training in the system, collecting and entering all data, and providing training to other staff within their organisations on reporting incidents. Organisations self-selected whether to trial UPLOADS-ST or UPLOADS-Lite based on the initial training session. Those that elected to participate in the UPLOADS-Lite trial identified at baseline that they did not want to do the full contributory factor analysis as part of UPLOADS-ST, usually because they felt they did not have enough incidents to warrant that effort.

This paper reports findings from the post-trial survey of 33 registered organisational users of UPLOADS-ST and 11 registered organisational users of UPLOADS-Lite. The proportion of all organisations that these 44 users represents is unknown, but the trial did involve all of the major national peak bodies, and some state-based organisations, that are driving this industry. The organisations that participated in the trial were self-selected from a broad invite to the sector from the research team. There is therefore a potential sampling bias present in that only organisations with a strong desire to improve safety, or those that did not like or have an existing incident reporting system, participated.

An online survey was administered to representatives of organisations that had used the system to ask them about their views about the system they used. Respondents were the nominated representative of the led outdoor activities organisation participating in the trial. The survey captured information on respondent demographics, experiences with the system, views about the system’s usability and sustainability, beliefs about the security and utility of the data. It was based on the previous survey successfully used in the pilot study to obtain views on use of the initial UPLOADS-ST system (Goode et al., 2018; Goode et al., 2015). The survey was tailored slightly for users of UPLOADS and UPLOADS-Lite, to reflect the relevant system design, and most items were rated on a 6-point Likert scale (unsure, strongly disagree, disagree, neutral, agree, strongly agree).

Because of the nature of the survey responses, and the relatively small numbers of survey respondents, results are presented as the number of responses and the disagree/strongly disagree, agree/strongly agree, and unsure/neutral, response categories were combined. There were insufficient numbers in each group to enable robust statistical comparisons across the two-systems so only descriptive findings are presented.

## Results

The overall survey response from UPLOADS-ST users was *n* = 18 from 33 participating organisations, of whom 14 responded that they used the UPLOADS-ST to enter data during the trial. From an initial 11 organisations who signed-up to use UPLOADS-Lite, six responded to the survey but only four indicated that they entered any data in the system. A mix of mid and large size organisations responded but the profiles of the responding organisations in both groups were similar in terms of size and focus. Respondents were typically male, held both a managerial position and led outdoor activities within their organisation, had 0–5 years’ experience in the led outdoor activities sector, held outdoor qualifications, and were the system administrator for their organisation at the start of the trial (Goode et al., n.d.). There was no information on the characteristics of the non-responders.

Table 2 summarises the respondents’ views about the system. Only four of the UPLOADS-ST users stated that the system was easy to use, and only three agreed that it was easy to enter incident report data. In contrast, the majority of UPLOADS-LITE users who tried the system considered that it was easy to use (*n* = 3) and entering data was easy (*n* = 3). The majority of UPLOADS-ST users and all UPLOADS-Lite users agreed/strongly agreed that the security and privacy of the systems were adequate for organisations and led outdoor activities participants.

**Table 2:** The views of UPLOADS-ST and UPLOADS-Lite users in relation to the specific system data collection, storage and reporting attributes

	UPLOADS-ST Users				UPLOADS-Lite Users			
	No. of responses	Disagree/strongly disagree	Neutral/Unsure	Agree/strongly agree	No. of responses	Disagree/strongly disagree	Neutral/ Unsure	Agree/strongly agree
Usability								
The training provided on reporting incidents is sufficient^	13	5	1	7	n/a *	–	–	–
Incident reports contain all relevant details*	13	3	2	8	4	0	1	3
Software tool is easy to use*	15	5	6	4	4	1	0	3
Entering incident reports is easy*	15	7	5	3	4	1	0	3
Entering participation data is easy*	15	1	6	8	4	1	0	3
Incident severity scale is logical*	15	3	3	9	4	0	1	3
Security and privacy								
Security and privacy is adequate for staff*~	14	0	3	11	4	0	0	4
Security and privacy is adequate for participants~	14	0	4	10	4	0	0	4
Sustainability								
Committed to contributing to system on ongoing basis#	17	1	6	10	6	1	1	4
Awareness to report incidents or near misses has improved*	13	2	4	7	6	4	1	1
If I enter data into UPLOADS I don’t need to enter elsewhere#	17	13	2	2	6	3	1	2
Entering reports is a good use of staff time and resources#	17	3	7	7	6	1	2	3
Utility for the sector as a whole								
6 monthly reports sufficient	12	0	1	11	6	0	0	6
First report easy to understand	12	2	3	7	6	0	1	5
Information provided was useful for my organisation	12	2	3	7	6	0	1	5
Information provided helped my organisation reduce incidents and near misses	12	4	7	1	6	1	5	0
In the long term, reports from the National Incident Dataset will help make sector safer	16	0	4	12	6	0	0	6

*question not relevant to UPLOADS-Lite users

A majority of respondents (ten for UPLOADS-ST, four for UPLOADS-Lite) indicated they were committed to ongoing data reporting through the system. Seven of the UPLOADS-ST users considered that their organisation’s awareness of the need to report data had improved but only one UPLOADS-Lite user felt the same over the 12 months. Many users of both systems indicated that they also needed to enter their data into other data systems for their organisation’s needs, with the suggestion that this added to their administrative burden. Less than half of respondents (seven UPLOADS-ST, three UPLOADS-Lite) considered that entering data reports was a good use of staff time and resources. Despite these perceived difficulties with the two systems that had been trialled, the vast majority of respondents (12 UPLOADS-ST, six UPLOADS-Lite) believed that having a national incident collection and reporting would make the lead outdoor activity domain safer.

## Discussion

Evaluation of injury data collection and reporting systems is essential to obtain feedback about their overall operation, determine whether their purposes are met, improve their performance and ensure their effectiveness (Ekegren et al., 2014; Bromley et al., 2018; Kerr et al., 2014; Calba et al., 2015; Romaguera et al., 2000; Ekegren et al., 2016; German et al., 2001). Importantly, the attributes of such systems need to be considered from the viewpoints of the end-users who need to use them (Bromley et al., 2018; Auer et al., 2011). This study presents the views of practitioners from the participating organisations in relation to the use of one of two reporting systems, UPLOADS-ST and UPLOADS-Lite, designed for the reporting of incident and injuries in the led outdoor activities sector.

Considering the usability of the systems, more UPLOADS-Lite than UPLOADS-ST users agreed that the system’s software interface was easy to use and that it was easy to enter data. This is not surprising given that UPLOADS-Lite was designed specifically as a less-resource intensive and easier to use version of UPLOADS-ST.

Regarding sustainability of both UPLOADS-ST and UPLOADS-Lite as an approach for ongoing surveillance, the majority of users indicated that their organisation was committed to maintaining data collection and that incident reporting was a good use of staff time and resources. However, the requirement to report adequate information on incidents to enable meaningful analysis and identification of contributory factors across the system needs to be balanced with the workload imposed (Evans et al., 2006). On this aspect, there was stronger support for UPLOADS-Lite. The data reported via UPLOADS-Lite did not provide sufficient information for the contributing organisations to undertake detailed analyses of incidents but those users were still keen for their data to contribute to a national incident database for these purposes and for an external agency to undertake the detailed incident analysis. A key challenge for injury surveillance and incident reporting generally is the development of systems that incur a low data collection burden yet still provide sufficient data to enable informative analyses.

A number of barriers to the sustainability of the systems were also identified. Specifically, the majority of organisations reported they still needed to enter the same data into their other organisational systems, affecting the administrative burden of their roles. In such circumstances, the requirements of externally based surveillance systems are likely to be considered less important to an organisation’s operations and this could influence data completeness and use of the system. A related issue was the lack of resources at the organisational levels available to enter reports and analyse the incident data collected. This could be at least partially due to the requirement to report incidents through multiple channels. Taken together, these three factors pose a significant threat to the sustainability of both the UPLOADS-ST and UPLOADS-Lite systems long term. It will also be important that a strong socio-political environment for the entire led outdoor activity sector provides additional support and incentives for organisations to contribute incident reports.

The utility of collected data is a key factor in sustaining reporting efforts over time (Ekegren et al., 2014; Mitchell et al., 2009; Bromley et al., 2018; Wu et al., 2008). Responses to the survey suggested the 14 organisations using UPLOADS-ST were making little direct use of the data they had been collecting, despite the fact that the system had been designed specifically to address industry-defined needs for such information (Salmon et al., 2017; Goode et al., 2014). It is possible that some of this was due to a perception that a particular participating organisation rarely experienced adverse incidents in its operations and this could have led to a belief that the reporting system was not relevant to them. In addition, it may be that this reflects a knowledge gap in terms of how to analyse and interpret multiple incident data sets. It is likely that organisations will require further education to increase their understanding of the value of reporting analysed data for their organisations, across all levels of risk, and ongoing guidance on how to analyse the incident data and contributory factors, and develop appropriate incident and injury prevention strategies (Salmon et al., 2010; Salmon et al., 2014; Goode et al., 2016b).

There are some limitations to the retrospective self-reported nature of this study. No independent assessment of the system performance and attributes could be made. As our focus was on the user experiences of these, the survey design was appropriate but could have been enhanced with the collection of more qualitative and open-ended responses. Non-responder bias cannot be excluded, as information was not collected on non-responders and only 55% of all organisations involved in the trial responded to the post survey. Not all responders answered every question because they had not all implemented the incident reporting within their organisation during the 12-months. It is possible that some non-responding organisations struggled to allocate internal resources to report injuries and incidents, and had either not implemented their UPLOADS system or had difficulty in completing the post-trial survey. Despite being anonymous, respondents may also have provided more socially acceptable responses for fear of identification, especially as they were responding on behalf of an organisation. Only one person from each organisation completed the survey, and other people from the same organisation may have had different experiences of the system they trialled. However, this possibility was minimised because the survey respondent was generally the major UPLOADS administrator for that organisation, and so would be most familiar with it. The sample size of respondents was not large and so the results may not be able to be generalised to all led outdoor activity providers, though the participants were from all organisation types and sizes across Australia. There may also be potentially important barriers towards the system use that were not included in the survey, which was designed to be succinct and not over burdensome for the respondents. Nonetheless, this study offers a foundation for future monitoring and implementation of the UPLOADS-ST and UPLOADS-Lite systems and has implications for the implementation of injury and incident surveillance systems more widely. Future research is warranted to determine the barriers and motivators of end-users towards the use of incident reporting systems, as a first step towards being able to increase end-user engagement with them into the future.

## Conclusions

The capacity of any surveillance system to describe patterns of incidents and their contributory factors accurately is critical in order to develop effective incident prevention strategies (Finch, 1997; Calba et al., 2015; Ekegren et al., 2016). It is therefore essential that reporting systems correctly report the occurrence of incidents and associated injuries, as well as their contributory factors. Evaluation of the effectiveness of incident reporting systems is therefore critical. Guidelines for evaluation of a good surveillance system, include a broad selection of attributes (German et al., 2001). These need to be adapted for specific situations and tailored to each evaluation. While data reporting systems have the potential to provide key insights into injuries and incidents to inform their prevention, they rely on organisations using them. Their end users’ experiences and the resources of contributing organisations, in turn, influence this. Identified barriers to the sustainability of both the UPLOADS-ST and UPLOADS-Lite systems included other reporting responsibilities within organisations, and a lack of infrastructure available to assist with entering data reports and analyse the incident data collected required by UPLOADS-ST. While surveillance systems have the potential to provide key insights into injuries and incidents to improve risk management, this study illustrates that their use by relevant sector stakeholders is highly dependent on users’ experiences and the resources of contributing organisations. Organisations can vary considerably in terms of their commitment to data collection and reporting incidents and thus their capacity to engage in such activities. These activities are crucial requirements of a systems-based approach to prevention (Goode et al., 2018) and require ongoing resourcing. Although development of UPLOADS was driven by a cohort of led outdoor activity stakeholders, previous incident reporting systems in this domain, such as the NZ National Incident Database (Salmon et al., 2014), have not had sustained success because of a lack of continuing funding to support ongoing training and data collection, analysis, and dissemination functions. Future efforts are needed to further increase buy-in to the systems approach in the led outdoor activity setting, and increasing the direct value and worth of incident reporting, whilst also reducing the administrative burden of effective data collection systems.

## Data Availability

Not applicable.

## Abbreviations

UPLOADS: Understanding and Preventing Led Outdoor Accidents Data System
UPLOADS-Lite: Modified version of the Understanding and Preventing Led Outdoor Accidents Data System Software Tool
UPLOADS-ST: The Understanding and Preventing Led Outdoor Accidents Data System Software Tool
